# An MRI Study of Neurovascular Restorative After Combination Treatment With *Xiaoshuan* Enteric-Coated Capsule and Enriched Environment in Rats After Stroke

**DOI:** 10.3389/fnins.2019.00701

**Published:** 2019-07-09

**Authors:** Yu Zhan, Man-Zhong Li, Le Yang, Xue-Feng Feng, Qiu-Xia Zhang, Nan Zhang, Yuan-Yuan Zhao, Hui Zhao

**Affiliations:** ^1^School of Traditional Chinese Medicine, Capital Medical University, Beijing, China; ^2^Beijing Key Lab of TCM Collateral Disease Theory Research, Beijing, China; ^3^Medical Imaging Laboratory of Core Facility Center, Capital Medical University, Beijing, China

**Keywords:** *Xiaoshuan* enteric-coated capsule, enriched environment, magnetic resonance imaging, neurovascular restoration, stroke

## Abstract

*Xiaoshuan* enteric-coated capsule (XSEC) is a Chinese medicinal compound widely used for treatment of ischemic cerebrovascular diseases. Enriched environment (EE) is an effective rehabilitative protocol designed to enhance sensorimotor, cognitive and social stimulation. This study aimed to apply magnetic resonance imaging (MRI) to non-invasively assess whether EE could augment the therapeutic benefits of XSEC on post-ischemic neurovascular remodeling. Male Sprague–Dawley rats were subjected to permanent middle cerebral artery occlusion (MCAO) and treated with XSEC and EE alone or combination for 30 consecutive days. Beam walking test and Morris water maze (MWM) test were performed to evaluate motor and cognitive function, respectively. Multimodal MRI was applied to examine alterations to brain structures, intracranial vessels, and cerebral perfusion on the 31st day after MCAO. Double-immunofluorescent staining was used to evaluate neurogenesis and angiogenesis. Western blot and RT-PCR were used to detect the expressions of vascular endothelial growth factor (VEGF), angiopoietin-1 (Ang-1), angiopoietin-2 (Ang-2), and the axon guidance molecules. Combination therapy with XSEC and EE significantly reduced cystic volume compared with XSEC and EE monotherapies. In line with this, combination treated rats performed better in the beam walking test and exhibited improved spatial memory in the probe trial of the MWM. Moreover, XSEC and EE combination treatment improved cerebral blood flow (CBF), amplified angiogenesis and upregulated VEGF protein levels. This proangiogenic effect was consistent with the increased progenitor cell proliferation and neuronal differentiation in the peri-infarct cortex and striatum. Specifically, the combined therapy of XSEC and EE markedly increased the Netrin-1 and Robo-1 protein expression levels compared with vehicle group, while no difference was observed between XSEC or EE monotherapy and vehicle group. Together, these findings indicate that the combination of XSEC and EE benefits neurovascular reorganization. This correlates with restoration of CBF, promotion of neurogenesis and angiogenesis, and activation of the intrinsic axonal guidance molecules, thereby facilitating greater physical rehabilitation after ischemic stroke.

## Introduction

Ischemic stroke is the leading cause of long-term adult disability worldwide. The neurovascular dysfunction following a period of ischemia causes cell death, brain atrophy, and may lead to chronic disability with lasting cognitive and motor disorders (Chu et al., 2012; Povlsen et al., 2016). Because neuroprotective therapies targeting individual pathogenic components of the ischemic cascade failed to show benefits in clinical trials, coordinated and multifunctional therapeutic approaches aiming to safely boost neurovascular repair processes during the post-stroke convalescence period are needed (Alokaily et al., 2018).

*Xiaoshuan* enteric-coated capsule (XSEC) is a novel preparation of the *Buyang Huanwu* Decoction (BYHWD), a traditional Chinese medicinal formula with a long history of treating post-stroke impairments (Li Z. et al., 2016). XSEC is authenticated and standardized based on marker compounds in the Chinese Pharmacopeia (Chinese Pharmacopoeia Commission, 2015), and is approved for treating ischemic stroke by the China Food and Drug Administration. XSEC has multi-target neurovascular protective effects on ischemic stroke, including restoring cerebral blood flow (CBF), preserving the neurovascular unit, and improving neuronal/glial metabolism (Zhang et al., 2016; Li M.Z. et al., 2018).

Enriched environment (EE) refers to housing conditions that increase opportunities for physical, intellectual, and social activity relative to standard housing conditions (Pusic et al., 2016). Substantial data indicate that housing in an EE results in positive neurological rehabilitation effects against brain insult in animal models, and has recently been translated into clinical practice (Rosbergen et al., 2017). Given the benefits of EE as a rehabilitative intervention after brain injury, the therapeutic effects of XSEC on post-ischemic neurovascular remodeling may be augmented by priming the brain with EE to maximize post-ischemic recovery. Magnetic resonance imaging (MRI) can be used to non-invasively monitor the hemodynamic and structural status of the living brain (Fisher et al., 2018). Accordingly, we used MRI to monitor hemodynamic changes and structural abnormalities after ischemic injury and specifically investigated the therapeutic effects of XSEC or EE monotherapy and combined therapy on neurovascular restoration in cerebral ischemic rats.

Many studies have shown that BYHWD or EE can boost endogenous repair mechanisms, such as angiogenesis and neurogenesis (Qu et al., 2014; Yang J. et al., 2015; Zhang et al., 2017; Wu et al., 2018). Ample evidence has shown that the coordinated action of attractive and repulsive extracellular axon guidance molecules, including Slits and Netrins, have an important role in the process of angiogenesis and neurogenesis except their role in axon pathfinding (Dumpich and Theiss, 2015; Kaneko et al., 2018). Netrin-1 binding to its receptor, deleted in colorectal cancer (DCC), is primarily known as attraction axonal growth cones to provide key guidance cues for axon outgrowth, axon orientation and neuronal migration during development (Serafini et al., 1994; Hong et al., 1999). In recent years, Netrin-1 has been demonstrated as a potent vascular mitogen that stimulates proliferation, migration, and tube formation in endothelial and vascular smooth muscle cells (Park et al., 2004). In contrast, Slit-2 was originally identified as a repulsive guidance cue on developing axons (Ypsilanti et al., 2010) and was later found to be pivotal in different types of cell migration (Whitford et al., 2002). Previous reports suggested that Slit-2 may play critical role in both physiological and pathological angiogenesis through its Robo-1 receptor expressed by both endothelial cells and pericytes (Li et al., 2015; Liu et al., 2018).

Therefore, we examined the impact of XSEC or EE monotherapy and their combination on angiogenesis and neurogenesis. We specifically detected the expression levels of axon guidance molecules Netrin-1 and Slit-2, and their receptors DCC and Robo-1 to determine whether these molecules are involved in poststroke neurovascular remodeling. These studies have important implications for the restorative use of XSEC and EE, and our results could provide a basis for priming the brain with EE to amplify the effects of XSEC treatment on stroke.

## Materials and Methods

### Animals and Drugs

Adult male Sprague–Dawley rats (aged 8 weeks, weighing 300–320 g) were purchased from Vital River Laboratory Animal Technology Co., Ltd. (Beijing, China). All rats were housed under 12-h/12-h light/dark cycles at 22 ± 1°C with food and water available. All experimental procedures were performed with protocols that were designed in accordance with the National Institute of Health Guide for the Care and Use of Laboratory Animals, and approved by the Capital Medical University Animal Ethics Committee (Permit Number: AEEI-2018-052).

*Xiaoshuan* enteric-coated capsule (batch no. 20170706) was obtained from Sanmenxia Sinoway Pharmaceutical Co., Ltd. (Henan, China) and approved by the Chinese State Food and Drug Administration (CFDA). The ingredients of XSEC were quality controlled as previously described (Zhang et al., 2016). XSEC was dissolved in physiological saline solution to a concentration of 28 mg/mL before experiment.

### Environmental Manipulation

For EE, animals were housed together in a large cage (90 cm long × 75 cm wide × 50 cm high) contained various toys (e.g., different shaped tubes, plastic tunnels, climbing ladders, balance beam, etc.). The subjects were changed every 2 days to keep novelty (Jiang et al., 2016). In addition, the EE provided enhanced social stimulation, 10–12 rats which included XSEC-treated and vehicle-treated were housed together. The environmental manipulation was administrated 30-days, 12 h per day (8:00 pm–8:00 am). For standard environment (SE), animals were housed in sets of three in standard cages (40 cm long × 30 cm wide × 20 cm high) with only food and water.

### Focal Cerebral Ischemic Model and Experimental Design

Focal cerebral ischemia was induced in rats by intraluminal occlusion of the right middle cerebral artery (MCAO) as previously described (Longa et al., 1989). Briefly, rats were anesthetized with isoflurane (5% for induction and 2% for maintenance) vaporized in N_2_O/O_2_ (70/30). Through a midline neck incision, the right common carotid artery (CCA), external carotid artery (ECA), and internal carotid artery (ICA) were exposed. The ECA and ICA were temporarily clamped using microsurgical clips. A 4-0 monofilament nylon suture (Beijing Sunbio Biotech Co Ltd., China) was inserted through the right ECA and gently advanced into the lumen of the ICA to occlude the origin of the middle cerebral artery (MCA).

Rats with successfully induced MCAO showing rotational asymmetry and dysfunctional limb placement (Yu et al., 2013) were randomly divided into four groups: (1) vehicle group: MCAO rats were administered with saline and housed in standard condition;(2) EE group: MCAO rats were administered with saline and housed in enriched environment; (3) XSEC group: MCAO rats were administered with XSEC and housed in standard condition; (4) XSEC + EE group: MCAO rats were administered with XSEC and housed in enriched environment. Sham-operated rats that underwent the same surgery but no occlusion procedure was grouped into the control group. In the control group, rats were administered with saline and housed in standard condition. XSEC was administered at 140 mg/kg by gavage once daily for 30 days starting at 24 h after MCAO. The animals in vehicle, EE and control group were given saline at 5 ml/kg in the same manner. Determination of the doses of XSEC used in the present study was based on our previous study which indicated that the administration of XSEC at 140 mg/kg of body weight improves cognitive function and cerebral hemodynamics in a chronic cerebral hypoperfusion rat model (Li M.Z. et al., 2018). Rats treated with EE were housed in the EE cages at 8:00 p.m. each day and returned to the standard cage at 8:00 a.m. the following day for a period of 30 days after MCAO.

In the neurobehavioral experiment (study 1), 50 rats (*n* = 9–11 per group) were used for the beam walking test on postoperative days 3, 7, 15, 20 and 25, and for learning and memory test in the Morris water maze (MWM). In the MRI and histological study (study 2), another 40 rats (*n* = 8 per group) underwent sequential MRI imaging and histological study without undergoing neurobehavioral experiments. Several studies have shown that behavioral training and testing may influence neuroplasticity (Gould et al., 1999; Engelmann et al., 2006; Lerch et al., 2011; Sumiyoshi et al., 2014). Recent study in rats showed that training in the MWM induced structural modifications and rapid changes in diffusion MRI indices (Hofstetter and Assaf, 2017). Rats were randomized to the treatment conditions. The order of the groups for testing was randomized based on a random number list to avoid experimenter bias while making observations (random number sequence was generated by: http://www.99cankao.com/numbers/random-number-generator.php). All experiments were performed in a blinded manner.

### Magnetic Resonance Imaging Protocols

Magnetic resonance imaging measurements were performed on the 31st day after MCAO using a 7.0T Bruker animal MRI scanner (Bruker, pharma Scan, Germany). Rats were anesthetized with isoflurane (5% for induction and 2% for maintenance). During the MRI scan, the rectal temperature of rats was kept at 37 ± 0.5°C with a feedback-controlled water bath system, and respiration was monitored throughout the experiment.

### T2 Relaxometry Mapping

T2 relaxometry mapping was performed to evaluate tissue injury using a multi-slice multi-echo sequence. The cystic volume, ventricular volume and hemispheric volume were calculated by multiplying the total cystic, ventricular, or hemispheric areas, respectively (bregma -3.2 to 1.2 mm), by the slice thickness. The parenchymal volume was then obtained by subtracting the ventricular volume from the hemispheric volume (Ding et al., 2010). The ratio of the parenchymal volume was calculated as the ipsilateral value relative to the contralateral. The 3D reconstruction of the cystic tissue was achieved by 3D Slicer software^1^.

#### Magnetic Resonance Angiography

Three-dimensional time-of-flight (TOF) magnetic resonance angiography (MRA) was applied to determine the status of the circle of Willis by using a fast- low- angle shot sequence. The data set images and maximal intensity projection (MIP) of MRA were acquired by Paravision version5.1 software (Bruker, Pharmascan, Germany). The vascular signal intensities in the anterior cerebral artery (ACA), MCA, ICA, and posterior cerebral artery (PCA) were obtained (Kara et al., 2012).

#### Arterial Spin Labeling

Arterial spin labeling (ASL) was carried out to monitor regional CBF with an echo-planar imaging fluid-attenuated inversion recovery (EPI-FLAIR) sequence (Zhang et al., 2018). Regions of interest (ROI) were first placed in the bilateral cortex and striatum on the T1 image and then copied to CBF map (bregma -0.2 mm) to acquire corresponding CBF. The relative CBF was further calculated as the ipsilateral CBF divided by the contralateral CBF (Yang Y. et al., 2015).

### Tissue Processing

At the end of MRI scanning, four rats from each group were deeply anesthetized and perfused transcardially with 0.9% saline followed by 4% paraformaldehyde in 0.1 m/L phosphate-buffered solution (PBS, pH 7.4). The brains were removed and post-fixed in the same fixative at 4°C overnight. Brain blocks located -0.4 to 0.4 mm relative to bregma were cut, processed, and embedded in paraffin. A series of 5 μm-thick coronal brain sections were sliced from the paraffin block for immunostaining.

The rest of the rats (four rats per group) were deeply anesthetized and sacrificed by decapitation. The brains were quickly dissected and the peri-ischemic tissues were separated for qRT-PCR and western blot analysis according to a previously described method (Ashwal et al., 1998).

### Immunofluorescence Staining

Double-label immunofluorescence staining was performed as previously described (Zhao et al., 2016). Briefly, sections were stained with primary antibodies to rabbit anti-CD31 (diluted 1:100; Abcam, cat. No. ab222783) and mouse anti-NG2 (diluted 1:100; Abcam, cat. No. ab50009) or to rabbit anti-Ki67 (diluted 1:800; Abcam, cat. No. ab16667) and mouse anti-Map-2 (diluted 1:3500; Abcam, cat. No. ab32454) or to rabbit anti-Ki67 and mouse anti-GFAP (diluted 1:800; Millipore, cat. No. MAB360), followed by incubation in species- and isotype-appropriate Alexa 488 and 594 secondary antibodies. The sections were then mounted onto slides, cover slipped with DAPI. Sections were visualized by Leica DMI6000 (TCS SP5, Leica, Germany) or by a digital camera connected to a fluorescence microscopy (Nikon 80i, Nikon, Tokyo, Japan). For the quantitation of angiogenesis, CD31^+^, NG2^+^, and CD31/NG2^+^ cells were counted in four randomly selected fields of the peri-infarct cortex and striatum. Data were expressed by the average number of cells per mm^2^. For neurogenesis, the amount of Ki67^+^, Map-2/Ki67^+^ and GFAP/Ki67^+^ co-expressed cells within the peri-infarct cortex and striatum were measured. The percentage of Map-2/Ki67^+^ or GFAP/Ki67^+^ co-expressed cells were estimated by division the number of double-labeled cells to the total number of Ki67^+^ cells (Santilli et al., 2010). All analyses were performed in a blinded manner.

### qRT-PCR

Total RNA was extracted from the peri-infarct tissues using RNAprep pure Tissue Kit (Tiangen, China, cat. No. DP431) and reverse transcribed into cDNA using FastQuant RT Kit (Tiangen, China, cat. No. KR106). RT-PCR was performed on a CFX Connect^TM^ Real-Time PCR Detection System (Bio-Rad) using SuperReal PreMix Plus (Tiangen, China, cat. No. FP205). The primer sequences were presented as follows (forward and reverse, respectively): VEGF, 5′-GCACGTTGGCTCACTTCCAG-3′ and 5′-TGGTCGGAACCAGAATCTTTATCTC-3′; Ang-1, 5′-ACCGTGAGGATGGAAGCCTAGA-3′ and 5′-AATGAA CTCGTTCCCAAGCCAATA-3′; Ang-2, 5′-CATGTCTAACGCCGTGCAGAG-3′ and 5′-GATCATCACAGCCGTCTGGTTC-3′; Netrin-1, 5′-TGCCAAAGGCTACCAGCAGA-3′ and 5′-GAAGCCTTGCAGTAGGAGTCACAG-3′; DCC, 5′- GATGGAGGTTATTGGCCAGTTGA-3′ and 5′-GTGACCACGGTTATGACAAGCAG-3′; Slit-2, 5′-GCCAAGGTTCGACCTCAGACA-3′ and 5′-CGCCCTCGATAGAGTTCCACA-3′; Robo-1, 5′-GCAACATGAGTGCTGCTGTAA-3′ and 5′-AGCTGTGCCTTGGACTGGA-3′; β-actin, 5′-GGAGATTACTGCCCTGGCTCCTA-3′ and 5′-GACTCATCGTACTCCTGCTTGCTG-3′. The quantitative data were presented as relative expression according to the formula of 2^−Δ Δ *Ct*^. Actin was used as an internal standard (Chen J. et al., 2017).

### Western Blot Analysis

The peri-ischemic brain tissues were homogenized in RIPA buffer (Applygen, China, cat. No. C1053) containing 1% phenylmethanesulfonyl fluoride (Applygen, China, cat. No. A1100) and 1% protease inhibitor (Applygen, China, cat. No. P1260). Protein concentration was determined using a BCA protein assay kit (Applygen, China, cat. No. P1511). Equal amounts of proteins were separated on 10% SDS-polyacrylamide gels, and then transferred onto polyvinylidene difluoride (PVDF) membranes (Millipore, United States, cat. No. IPVH0010). Membranes were blocked with 5% non-fat milk or BSA for 1 h at room temperature and then incubated overnight at 4°C with the primary antibodies against the following proteins: VEGF (diluted 1:2000; Abcam, cat. No. ab46154), Ang-1 (diluted 1:2000; Millipore, cat. No. AB10516), Ang-2 (diluted 1:1000; Abcam, cat. No. ab125692), Netrin-1 (diluted 1:40000; Abcam, cat. No. ab126729), DCC (diluted 1:5000; Abcam, cat. No. ab125280), Slit-2 (diluted 1:60000; Abcam, cat. No. ab134166), Robo-1 (diluted 1:40000; Abcam, cat. No. ab85312), and GAPDH (diluted 1:40000; CWBIO, cat. No. NBL01c). After washing, membranes were incubated with secondary horseradish peroxidase-labeled anti-rabbit (diluted 1:20000; CWBIO, cat. No. CW0103S) or anti-mouse (diluted 1:20000; CWBIO, cat. No. CW0102S) IgG. Immunoreactive bands were visualized using the super ECL plus kit (Applygen, China, cat. No. P1050). X-ray films were scanned and analyzed by measuring the optical densities of the protein bands using ImageJ software.

### Behavioral Tests

The motor function of rats was evaluated by the beam walking test on postoperative days 3, 7, 15, 20, and 25. Before operation, the rats received three training trials for 3 days. Briefly, rats were habituated to walk on a wooden beam before ischemia induction. After MCAO, beam walking tests were conducted with the following scoring system (Sword et al., 2013): (1) unable to traverse the beam or place the affected hindlimb on the horizontal surface of the beam; (2) able to place affected hindlimb on the beam surface but unable to traverse; (3) traverse the beam while dragging the affected hindlimb; (4) traverse the beam and at least once places the affected hindlimb on the horizontal surface of the beam; (5) traverse the beam and use the affected hindlimb in less than half of its steps along the beam; (6) traverse the beam and use the affected hindlimb in more than half of its steps along the beam; (7) traverse the beam with no more than two foots lips.

Spatial learning and memory were assessed using a modified version of the MWM task on the 31st day after MCAO (Zhang et al., 2015). The MWM test was performed in a circular pool filled with water (18–20°C) and divided into four quadrants of equal surface area (quadrants I, II, III, and IV). The procedure consisted of 4 days of hidden platform test and 1 day of probe trial (Wang X. et al., 2016). In the hidden platform test, the platform was submerged 1.5 cm below the water surface in the middle of the quadrant I. Two trials comprised a session, and two sessions per day were performed with 1 h intervals for 4 consecutive days. For each trial, the rat was allowed to swim for at most 60 s before locating the submerged platform. If the rat failed to find the platform, it was gently guided to the platform and left on it for 10 s. A video camera connected to an image analyzer (JLBehv-MWMG, Jiliang Sofware Technology Co., Ltd., Shanghai) recorded latency and swim path length to find the platform. The probe trial was conducted at 24 h after the last hidden platform test to assess spatial memory capacity. The platform was removed from the maze and rats were allowed to swim for 30 s in the pool. The percentage of time spent by rats in the target quadrant (quadrant I) was calculated. The analysis of data was done by an experimenter blinded to the experimental conditions of the animals.

### Statistical Analysis

Data were presented as mean ± standard error of the mean (SEM). All data were analyzed using the SPSS 21.0 (SPSS Inc., United States) software. Statistical significance was determined when *P* < 0.05. Two-way ANOVA was conducted on data from MRI, Immunofluorescence staining, qRT-PCR, Western blot and probe test to determine the main effects of XSEC and EE and their interactive effects among vehicle, EE, XSEC and XSEC+EE rats. One-way ANOVA with *post hoc* Bonferroni correction was conducted to determine significance of differences between vehicle, EE, XSEC, XSEC+EE, and control rats. Data from the beam walking test and hidden platform test were statistically analyzed with two-factor repeated-measures ANOVAs (between-subjects factor: treatment; within-subjects factor: time). *Post hoc* comparisons using Tukey’s analyses were conducted. Statistical analyses were performed on data collected by experimenters blinded to the treatment conditions.

## Results

### Combination Treatment With XSEC and EE Reduced Lesion Volume and Cerebral Atrophy

**FIGURE 1:**
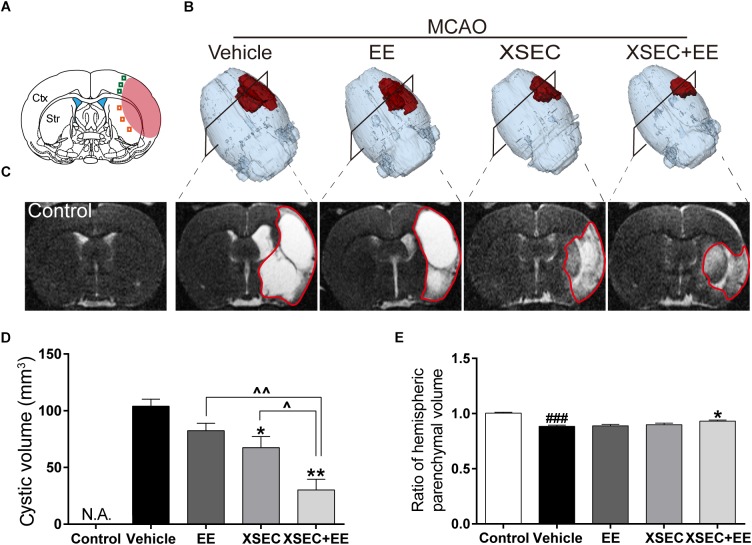
Effect of combination treatment with XSEC and EE on the tissue loss and injury in MCAO rats. **(A)** Schematic diagram of the ischemic brain. The red region represents infarct area. The green square represents peri-ischemic cortex region and orange for striatum. **(B)** Typical 3D reconstruction images of cystic tissue were achieved by 3D Slicer software (red represented the cystic volume). **(C)** Representative T2 maps of bregma –0.80 mm from various groups. Quantitative analysis of **(D)** cystic volume, **(E)** hemispheric parenchymal volume ratios (ipsilateral vs. contralateral). Data were presented as mean ± SEM, *n* = 8, ^###^*P* < 0.001 vs. control; *^∗^P* < 0.05 and *^∗∗^P* < 0.01 vs. vehicle; ^∧^*P* < 0.05 and ^∧∧^*P* < 0.01 vs. XSEC+EE.

T2 maps revealed a hyperintense signal in the MCA territory representing structural loss (Figure 1C). Two-way ANOVA revealed the main effect of XSEC [*F*_(1,31)_ = 24.635, *P* < 0.01] and EE [*F*_(1,31)_ = 9.755, *P* = 0.04] on reduced the cystic volume, but there was no interaction effect. *Post hoc* analysis revealed that vehicle rats significantly decreased hemispheric parenchymal volume ratio compared with control group (*P* < 0.001, Figure 1A,E). However, XSEC+EE group significantly increased the hemispheric parenchymal volume ratio compared to vehicle group (*P* < 0.05). Furthermore, combination treatment and XSEC monotherapy significantly reduced the cystic volume compared with vehicle group (*P* < 0.05 or *P* < 0.01, Figure 1B,D). Specifically, *post hoc* analysis showed cystic volume was significantly lower in combination treatment rats compared with XSEC or EE monotherapy (*P* < 0.05 or *P* < 0.01), strongly suggesting that the combination of XSEC and EE could reduce cerebral atrophy.

### Combination Treatment With XSEC and EE Improved Cerebral Perfusion

**FIGURE 2:**
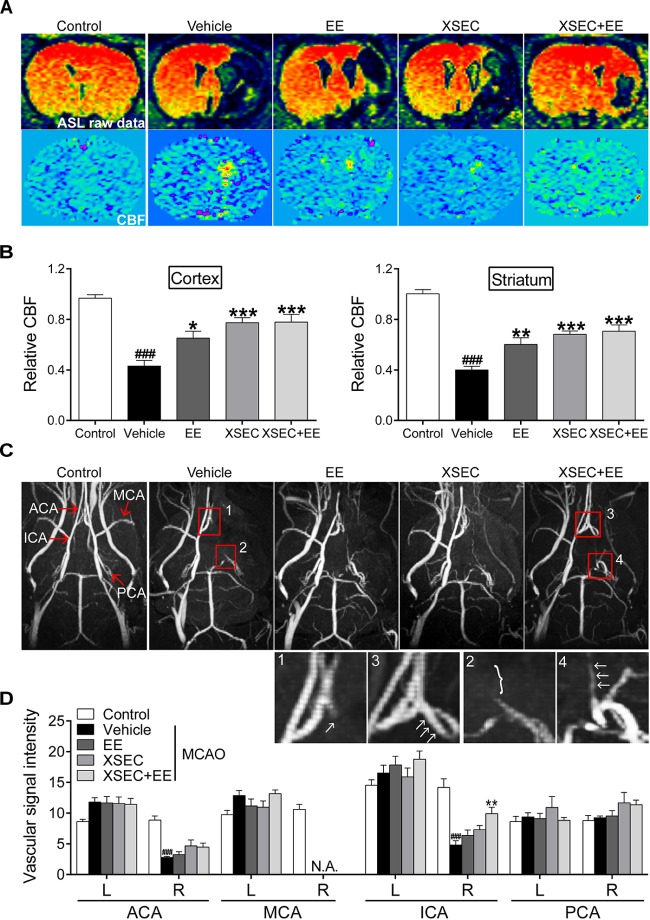
Effect of combination treatment with XSEC and EE on cerebral perfusion in MCAO rats. **(A)** Representative ASL raw data maps and CBF maps were obtained from various groups. **(B)** The analysis of result of the relative CBF in the ipsilateral cortex and striatum. **(C)** Representative axial MIP maps of MRA were obtained from various groups. The amplified ACA (1, 3) and ICA (2, 4) signal pictures were below. **(D)** Quantitative analysis of vascular signal intensity. ACA, anterior cerebral artery; MCA, middle cerebral artery; ICA, internal carotid artery; PCA, posterior cerebral artery. Data were presented as mean ± SEM, *n* = 8, ^###^*P* < 0.001 vs. control; *^∗^P* < 0.05, *^∗∗^P* < 0.01, and ^∗^*^∗∗^P* < 0.001 vs. vehicle.

Magnetic resonance angiography was carried out to examine the status of intracranial vessels. Axial MIP of MRA maps demonstrated absence signal of the right MCA. Two-way ANOVA revealed the main effect of XSEC [ipsilateral: ICA: *F*_(1,31)_ = 15.557, *P* < 0.01, ACA: *F*_(1,31)_ = 5.186, *P* = 0.031, PCA: *F*_(1,31)_ = 11.336, *P* = 0.02; BA:*F*_(1,31)_ = 7.783, *P* = 0.009] and EE [ipsilateral: ICA: *F*_(1,31)_ = 7.800, *P* = 0.09] on vascular signal intensity of the ipsilateral hemisphere. *Post hoc* analysis showed vehicle rats decreased signal intensity in the ipsilateral ACA and ICA compared to control rats (*P* < 0.001, Figure 2C,D). On the contrary, the signal intensities from the ipsilateral ICA were elevated by the combination of XSEC and EE treatment compared with vehicle group (*P* < 0.01).

Next, quantitative CBF imaging using ASL showed a drop in CBF on the ischemic side corresponding to the cortex and striatum after MCAO. Two-way ANOVA revealed a significant main effect for XSEC [cortex: *F*_(1,31)_ = 21.276, *P* < 0.01; striatum: *F*_(1,31)_ = 26.470, *P* < 0.01] or EE monotherapy [cortex: *F*_(1,31)_ = 4.938, *P* = 0.035] and a significant interaction effect [cortex: *F*_(1,31)_ = 4.482, *P* = 0.043; striatum: *F*_(1,31)_ = 7.796, *P* = 0.009] in relative CBF of the ipsilateral cortex and striatum. *Post hoc* analysis revealed a significant lower relative CBF in the right cortex and striatum of vehicle rats than control rats (*P* < 0.001, Figure 2A,B). However, rats received combination treatment and monotherapy with XSEC or EE significantly increased relative CBF of the ipsilateral cortical and striatal compared with vehicle rats (*P* < 0.05 or *P* < 0.01 or *P* < 0.001). Taken together, these data indicated that combination treatment facilitated the compensation of collateral vessels network to achieve functional perfusion, ultimately alleviating tissue damage after stroke.

### Combination Treatment With XSEC and EE Promoted Angiogenesis

**FIGURE 3:**
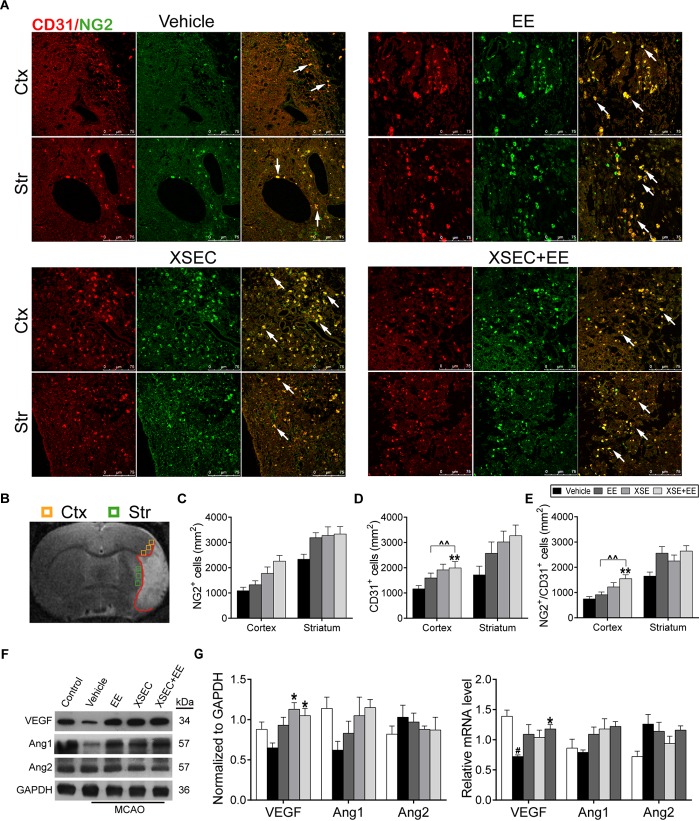
Effect of combination treatment with XSEC and EE on the angiogenesis in MCAO rats. **(A)** Representative confocal images of double immunofluorescent staining for CD31 and NG2 in the peri-infarct cortex and striatum (white arrows indicated co-located cells) of different groups. **(B)** Schematic diagram of the ischemic brain. The yellow square represents peri-ischemic cortex region and green for striatum that was dissected for immunofluorescence. **(C–E)** Quantitative data of the number of NG2^+^, CD31^+^ and NG2/CD31^+^ cells per mm^2^ in the peri-infarct cortex and striatum. **(F)** The representative image of western-blot analysis for VEGF/Ang-1/Ang-2 **(G)** The mRNA and protein expression of VEGF/Ang-1/Ang-2 were determined by RT-PCR and western blot analysis. Data are expressed as means ± SEM, n = 4 for each group. ^#^*P* < 0.05 vs. control; *^∗^P* < 0.05 and *^∗∗^P* < 0.01 vs. vehicle; ^∧∧^*P* < 0.01 vs. XSEC+EE.

To study the potential of combination treatment on angiogenesis, the number of NG2^+^ (a marker of pericytes), CD31^+^ (a marker of vascular endothelial cells) and NG2/CD31^+^ cells was calculated. Two-way ANOVA revealed that XSEC made main effect in NG2^+^ [cortex: *F*_(1,19)_ = 6.242, *P* = 0.016; striatum: *F*_(1,19)_ = 5.094, *P* = 0.029], CD31^+^ [cortex: *F*_(1,19)_ = 15.087, *P* < 0.001] and NG2/CD31^+^ cells [cortex: *F*_(1,19)_ = 15.043, *P* < 0.001]. And EE made main effect in NG2/CD31^+^ [striatum: *F*_(1,19)_ = 15.043, *P* < 0.001] cells. However, there was no interaction between XSEC and EE. *Post hoc* analysis revealed that the combination of XSEC and EE caused a markedly increase of CD31^+^ and NG2/CD31^+^ cell numbers in the peri-infarct cortex compared with vehicle group (*P* < 0.01, Figure 3A–E), indicating enhanced angiogenesis. Notably, the numbers of CD31^+^ and NG2/CD31^+^ cells in the perilesional cortex were significantly increased in XSEC + EE group compared with that of the EE group (*P* < 0.01).

Furthermore, angiogenesis-related factors including VEGF/Ang-1/Ang-2 were investigated by western blot and quantitative RT-PCR. Two-way ANOVA revealed a significant main effect for XSEC in VEGF protein [*F*_(1,19)_ = 12.230, *P* = 0.003] and Ang-2 mRNA [*F*_(1,19)_ = 8.300, *P* = 0.011] expression level. Combination treatment displayed a significant interaction between two monotherapy group in VEGF protein level [*F*_(1,19)_ = 4.509, *P* = 0.048]. *Post hoc* analysis showed the VEGF mRNA level was notably decreased in the vehicle group compared to the control group (*P* < 0.05, Figure 3F,G). Cotreatment with XSEC and EE significantly elevated the VEGF mRNA and protein levels compared with vehicle treatment (*P* < 0.05). In addition, XSEC monotherapy resulted in significant increased protein expression of VEGF compared to vehicle group (*P* < 0.05). On the other side, an increased expression of Ang-1 but a decreased expression of Ang-2 were observed in EE, XSEC, and XSEC+EE group, which was not significantly different compared to vehicle group.

### Combination Treatment With XSEC and EE Boosted the Endogenous Neurogenesis

**FIGURE 4:**
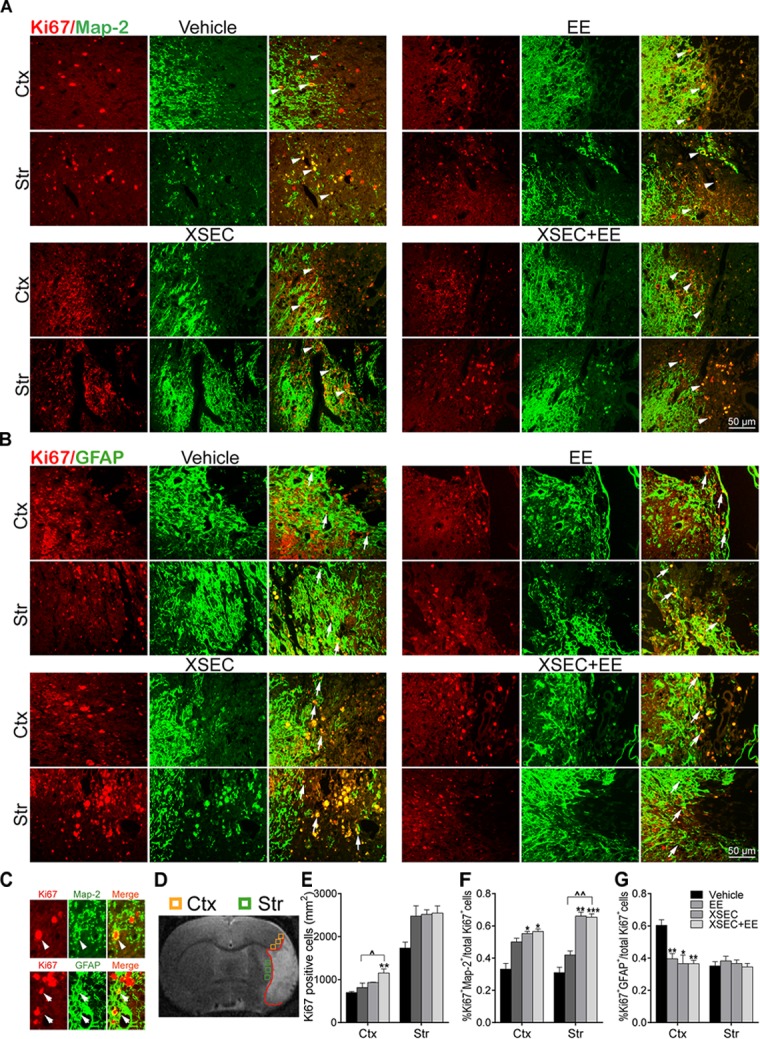
Effect of combination treatment with XSEC and EE on the endogenous neurogenesis in MCAO rats. **(A,B)** Typical photographs of double immunofluorescent staining for Map-2/Ki67 and GFAP/Ki67 in the peri-infarct cortex and striatum (white arrows indicated co-located cells). **(C)** Magnified images of co-expressed cells of Map-2/Ki67 ^+^ and GFAP/Ki67^+^ (arrowheads: Map-2/Ki67^+^ co-expressed cell; arrows: GFAP/Ki67^+^ co-expressed cell). **(D)** Schematic diagram of the ischemic brain. The yellow square represents peri-ischemic cortex region and green for striatum that wasdissected for immunofluorescence. **(E–G)** Quantitative data of the number of Ki67^+^ cells per mm^2^ and the percentage of Map-2/Ki67 ^+^ and GFAP/Ki67^+^ co-expression cells divide by total Ki67^+^ cells numbers in the peri-infarct cortex and striatum. Data were presented as mean ± SEM, *n* = 4, *^∗^P* < 0.05, *^∗∗^P* < 0.01, *^∗∗∗^P* < 0.001 vs. vehicle; ^∧^*P* < 0.05, ^∧∧^*P* < 0.01 vs. XSEC+EE.

To examine the effects of combinatorial treatment on cell proliferation, the number of Ki67^+^ cells was measured. Two-way ANOVA revealed the main effect of XSEC in Ki67^+^ cells numbers [cortex: *F*_(1,19)_ = 13.07, *P* = 0.003; striatum: *F*_(1,19)_ = 22.31, *P* < 0.001]. *Post hoc* analysis revealed that rats received cotreatment with XSEC and EE significantly elevated the number of Ki67^+^ cells in the peri-infarct cortex compared with vehicle group (*P* < 0.01, Figure 4E). Combination therapy rats also increased number of Ki67^+^ cells in the peri-infarct cortex compared with EE rats (*P* < 0.05).

Furthermore, the effect of XSEC+EE treatment on the differentiation of the proliferating cells was determined (Figure 4A–G). Two-way ANOVA revealed a significant main effect for XSEC in percentages of GFAP/Ki67^+^ [cortex: *F*_(1,19)_ = 11.86, *P* = 0.004] and MAP-2/Ki67^+^ cells [cortex: *F*_(1,19)_ = 9.86, *P* = 0.009; striatum: *F*_(1,19)_ = 46.38, *P* < 0.001]. EE monotherapy made main effect in percentages of GFAP/Ki67^+^ cells [cortex: *F*_(1,19)_ = 9.19, *P* = 0.008]. And there was a significant interaction in percentages of GFAP/Ki67^+^ cells [cortex: *F*_(1,19)_ = 4.86, *P* = 0.043]. *Post hoc* analysis revealed that combination therapy and XSEC monotherapies increased the percentages of MAP-2/Ki67^+^ cells in the peri-infarct cortex (*P* < 0.05) and striatum (*P* < 0.01 or *P* < 0.001). Furthermore, the percentages of MAP-2/Ki67^+^ cells were significantly increased in the XSEC+EE group compared to EE group. Surprisingly, rats received combination treatment and monotherapy with XSEC or EE exhibited significant reduction in the percentages of GFAP/Ki67^+^ cells of the ipsilateral cortex compared with vehicle group (*P* < 0.05 or *P* < 0.01, Figure 4G). These results suggested XSEC and EE combination amplified the cell proliferation, promoted neuronal differentiation in the peri-infarct areas.

### Combination Treatment With XSEC and EE Regulated the Expression of Netrin-1/DCC, Slit-2/Robo-1 in MCAO Rats

**FIGURE 5:**
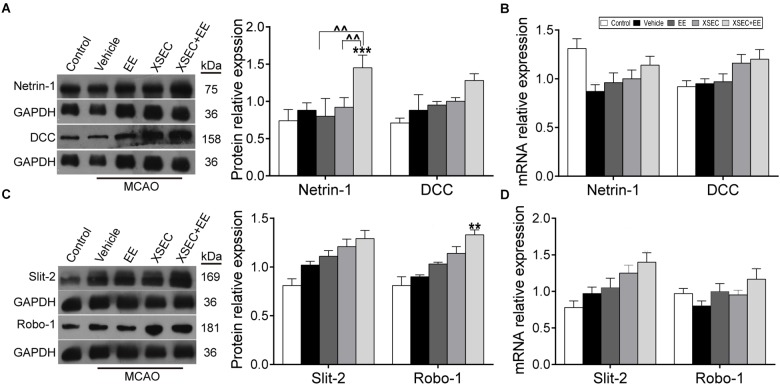
Effect of XSEC+EE on guidance factor in MACO rats. The representative image and western-blot analysis for Netrin-1/DCC **(A)** and Slit-2/Robo-1 **(C)** were showed on the left. The mRNA of Netrin-1/DCC **(B)** and Slit-2/Robo-1 **(D)** was determined by RT-PCR analysis. The protein level of Netrin-1/DCC and Slit-2/Robo-1 was quantified using GAPDH as the loading controls. Data are expressed as means ± SE, *n* = 4 for each group. *^∗∗^P* < 0.01 and *^∗∗∗^P* < 0.001 vs. vehicle; ^∧∧^*P* < 0.01 vs. XSEC+EE.

Attractive and repulsive extracellular axon guidance molecules play vital roles in the process of angiogenesis and neurogenesis (Figure 5A–D). Two-way ANOVA revealed a significant main effect for XSEC in DCC [*F*_(1,19)_ = 8.28, *P* = 0.009] and Slit-2 [*F*_(1,19)_ = 7.447, *P* = 0.013] mRNA level. XSEC also made main effect in Netrin-1 [*F*_(1,19)_ = 7.47, *P* = 0.015], Robo-1 [*F*_(1,19)_ = 17.376, *P* = 0.001], and Slit-2 [*F*_(1,19)_ = 4.740, *P* = 0.046] protein level. And EE made main effect in Netrin-1 [*F*_(1,19)_ = 23.15, *P* < 0.001] and Robo-1 [*F*_(1,19)_ = 6.035, *P* = 0.029] protein level. Moreover, there were positive interaction between XSEC and EE monotherapy in Netrin-1 [*F*_(1,19)_ = 4.54, *P* = 0.049] and Robo-1 [*F*_(1,19)_ = 6.035, *P* = 0.029] protein level. *Post hoc* analysis revealed that rats received XSEC and EE combination treatment significantly upregulated protein levels of Netrin-1 and Robo-1 compared with vehicle group (*P* < 0.01 or *P* < 0.001, Figure 5A,C). Furthermore, Netrin-1 protein level was significantly increased by combination treatment compared to monotherapy with XSEC or EE (*P* < 0.01).

### Combination Treatment With XSEC and EE Improved Neurological Function in MCAO Rats

**FIGURE 6:**
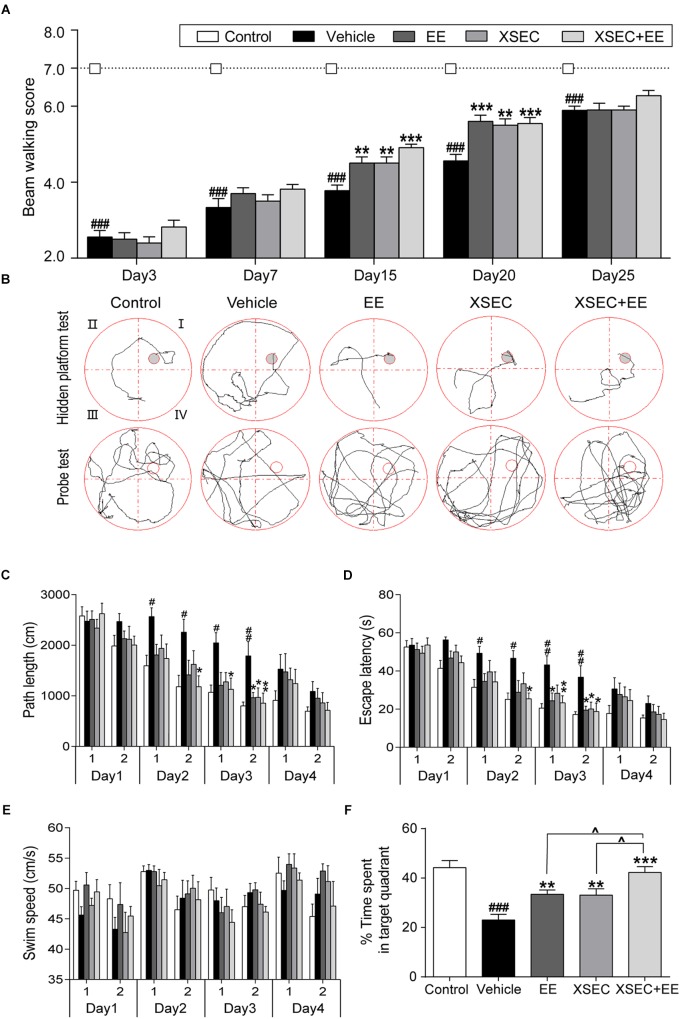
Effect of combination treatment with XSEC and EE on the neurobehavior function in MCAO rats. **(A)** Assessment of motor functions by beam walking. **(B)** Typical training traces of rats in hidden platform test (top panel) on second session of 4th day and typical traces in probe test (bottom panel). **(C)** Path length, **(D)** escape latency, and **(E)** swim speed of rats in the hidden platform test. **(F)** Percentage time spent by rats in the target quadrant in the probe trial. Data were presented as mean ± SEM, *n* = 9–11, ^#^*P* < 0.05, ^##^*P* < 0.01, and ^###^*P* < 0.001 vs. control; *^∗^P* < 0.05, *^∗∗^P* < 0.01, and ^∗∗∗^*P* < 0.001 vs. vehicle; ^∧^*P* < 0.05 vs. XSEC+EE.

The beam walk test was used to assess recovery of rats. Repeated measures of ANOVA showed considerable effects of time [*F*_(2,48)_ = 335.196, *P* < 0.001] and treatment [*F*_(2,48)_ = 232.048, *P* < 0.001] on the beam walk scores. Importantly, a time × treatment interaction was found on the beam walk scores of baselines [*F*_(2,48)_ = 23.265, *P* < 0.001]. These data suggested the motor function of MCAO rats recovered overtime and treated rats exhibited better motor recovery than vehicle rats. *Post hoc* analysis showed MCAO decreased the beam walk scores compared with the control group from 3rd day to 25th day. Specifically, Rats received combination treatment and monotherapy with XSEC or EE increased beam walk scores on 15th and 20th day after MCAO compared with vehicle group (*P* < 0.01 or *P* < 0.001, Figure 6A).

In the hidden platform test (Figure 6B), repeated measures of ANOVA revealed significant effects of time and treatment on the path length [time: *F*_(7,315)_ = 35.758, *P* < 0.001; treatment: *F*_(4,45)_ = 5.284, *P* = 0.001] and escape latency [time: *F*_(7,315)_ = 45.161, *P* < 0.001; treatment: *F*_(4,45)_ = 6.557, *P* < 0.001]. These results suggested rats gained spatial learning overtime and treated rats performed better than vehicle rats. *Post hoc* analysis showed that vehicle rats took longer time and path to find the hidden platform compared with control rats on the 2nd and 3rd day (*P* < 0.05 or *P* < 0.01, Figure 6C,D). XSEC+EE treatment rats displayed shorter escape latency and path length compared with the vehicle animals in the second session of 2nd day up to the 3rd day (*P* < 0.05 or *P* < 0.01). EE monotherapy rats significantly shortened the escape latency in 3rd day and cut the path length in second session of 3rd day compared with vehicle rats (*P* < 0.05). Rats in the XSEC group also displayed less length and time to locate the submerged platform in the second session of 3rd day compared with vehicle rats (*P* < 0.05). Swimming speed during 4 days was no significantly different between groups, indicating that no motor impairments influenced the acquisition of spatial learning (Figure 6E).

The probe test was used to assess memory retention (Figure 6B). Rats in the vehicle group spent less time in the original quadrant where the platform was previously situated, indicating impaired spatial memory. Two-way ANOVA revealed a significant main effect for XSEC [*F*_(1,39)_ = 11.556, *P* = 0.002] and EE [*F*_(1,39)_ = 24.895, *P* < 0.001, Figure 6F] in time spent by rats in the target quadrant, but there was no interaction effect. *Post hoc* analysis showed that vehicle rats decreased the time spent in the target quadrant compared with control group (*P* < 0.001). All the treatment markedly increased the time by rats spent in the target quadrant compared with vehicle group (*P* < 0.01 or *P* < 0.001). Notably, rats treated with XSEC+EE spent more time in the target quadrant compared with rats treated with either XSEC or EE (*P* < 0.05).

## Discussion

In the present study, we showed that combination treatment with XSEC and EE significantly reduced cerebral atrophy and promoted long-term neurological recovery up to 30 days after ischemia. Our data also showed that combination treatment induce a significant interactive effect in improved CBF and enhanced VEGF as well as down-regulation of Ki67-positive astrocytes after stroke. Meanwhile, axonal guidance signaling Netrin-1 and Robo-1 would be expected to participate in the beneficial effects of the combined actions of XSEC and EE on angiogenesis and neurogenesis in the ischemic brain after stroke.

T2 relaxometry mapping has been widely applied to delineate the tissue abnormality after ischemia (Qiao et al., 2001). Our results showed that the cystic volume was significantly increased and the hemispheric parenchymal volume was remarkably decreased in vehicle group. EE alone did not produce beneficial effects in restoring brain infarct volume, which is in agreement with previous studies (Kline et al., 2010; Leary et al., 2017). XSEC individually revealed main effect in decreased the infarct volume, but had no effect on hemispheric parenchymal volume. The data are consistent with our previous reports (Zhang et al., 2016). Remarkably, the combination treatment showed significant smaller cystic volume and larger hemispheric parenchymal volume compared with just a single intervention. On face value, this finding suggested that the combination treatment may provide additive benefits for the reduction in brain tissue loss in MCAO rats.

Both animal and clinical studies have shown that BYHWD can improve neurologic outcome after stroke (Hao et al., 2012). Moreover, many studies encourage EE for recovery of motor (Yang et al., 2017; Zhang et al., 2017) and cognitive functions (Wang L. et al., 2016; Gonçalves et al., 2018) following cerebral ischemic injury. Based on the above findings it is of particular interest to investigate whether the combined therapeutic potential of EE and chronic administration of XSEC would be greater than that of either treatment alone on recovery of motor and cognitive function. Our study showed that XSEC improved the recovery of motor function, spatial learning and memory in the probe trial of the MWM following ischemic stroke. Interestingly, rats that were housed in EE also showed improved functional outcomes in the absence of an effect on infarct volume. Our results are consistent with previous reports indicating that the EE-associated function recovery is not accompanied by reduced infarct volume (Janssen et al., 2010; Shono et al., 2011; Xie et al., 2013; Griva et al., 2017). Given that EE-associated induced functional recovery is not associated with infarct volume after ischemic stroke, others have suggested that the beneficial effects of EE are mainly related to the activation of endogenous restorative and regenerative mechanisms. Specifically, enhancement of synaptic plasticity accompanied with upregulation of neurotrophin expression and reorganization of neuronal networks in the remaining brain might contribute to behavioral improvement following post-injury environmental manipulations (Xie et al., 2013; Chen X. et al., 2017). Surprisingly, the combined therapy of XSEC and EE further enhanced spatial memory in the probe trial of the MWM compared to those with just a single intervention after ischemic stroke. Regarding beam walking test and hidden platform test, the combined therapy rats performed better versus vehicle controls, but the effects were not statistical different compared to monotherapy group. A possible interpretation regarding the lack of additive effects with the combination therapy may be that EE and XSEC independently confer significant benefit to motor function and spatial learning after ischemic stroke evaluated in this study. Previous studies also reported a similar ceiling effect that implementation of EE combined with other pharmacological agents did not reveal greater effects on neurobehavioral and cognitive after brain damage in the rats than a solitary treatment (Kline et al., 2010, 2012; Leary et al., 2017). Overall, despite the lack of an additive effect on spatial learning improvement, the combined therapy paradigm of EE and XSEC did confer greater protection against MCAO-induced tissue damage than either therapy alone.

Ischemic stroke is caused by interruption of the blood supply to some regions of the brain, and the restoration of cerebral circulation is a crucial role in the compensating for the detrimental effects in the ischemic injury (Liu et al., 2014). ASL perfusion imaging is an ideal imaging modality to determine dynamic cerebral blood changes. And TOF-MRA is capable of providing angiographic information (Besselmann et al., 2001). Using this technique, we assessed the separate and combined actions of XSEC and EE on long-term blood circulation following ischemic stroke. In the present study, MRA revealed absent signal in ipsilateral MCA and decreased signal intensity in the ipsilateral ACA and ICA of vehicle group rats. In addition, ASL perfusion imaging showed retrograde CBF within the MCA territory of the ischemic side. These MRI-based findings indicated poor collateral status following MCA occlusion as evidenced by vessel anatomy and retrograde recruitment of the collateral flow in the ipsilateral ischemic region in vehicle group rats. In contrast, combined treated rats exhibited augmented CBF in the ischemic cortex and striatum as well as increased signal intensity of the ipsilateral ICA on MR angiograms, suggesting augmented collateral flow through ICA to MCA territory of the ischemic side. It should be noted that combination treatment evoked significant interactive effects on relative CBF of the ipsilateral cortex and striatum in the ischemic brain after stroke. Specifically, XSEC treatment and EE individually also improved the relative CBF in the in the corresponding regions, but exhibited no significant signal intensity in the carotid artery system compared to vehicle group. These findings raised the interesting possibility that the beneficial effects of combination XSEC and EE toward collateral blood flow augmentation after ischemic stroke.

It has been previously demonstrated that a better collateral circulation and amplified CBF along the ischemic/hypoxic territory might provide a favorable environment for the activation of endogenous angiogenesis (Chen et al., 2014). At the same time, angiogenesis plays a critical role in maintaining regional cerebral blood flow in the subacute stage of cerebral ischemia (Virgintino et al., 2007; Liu et al., 2014). Thus, it cannot be excluded that the post-stroke angiogenesis might contribute to improve CBF in peri-infarct brain after XSEC and EE treatment. In light of this, the present investigation assessed the separate and combined actions of XSEC and EE on angiogenesis following ischemic stroke. Angiogenesis is defined as the formation of new blood vessels by newly generated endothelial cells. During the process of angiogenesis, the initial endothelial cell proliferation is important for vessel growth, and pericytes provide structural support to endothelial cells and guide the formation of capillary tubes during organization of the growing vessel wall (Fukushi et al., 2004; Bergers and Song, 2005). In this study, endothelial cells were identified by the pan-endothelial marker CD31, and the activated pericytes were confirmed through the specific marker NG2 proteoglycan, which tightly invest endothelial cells and initiate the angiogenic process to form an integral new vessel (Virgintino et al., 2007). EE has been shown to exert beneficial effects on ischemic stroke rats by inducing angiogenesis and modifying the expression of angiogenic growth factor (Zhang et al., 2017). According to our findings, combined actions of XSEC and EE could increase the number of the CD31-positive endothelial cells as well as NG2/CD31doublelabeled cells in the ischemic boundary zone of cortex compared with vehicle group, indicating combination treatment involved in the angiogenic activated state. These results, along with improving the blood supply to the peri-infarct tissue, likely reflected the capacity of XSEC or EE facilitating angiogenesis, which may help preserve CBF in peri-infarct brain tissue and f restore neurological function of the stroke animals.

Angiogenesis is a complex biological process regulated by a variety of angiogenic growth factors, which have been demonstrated to be crucial in the reciprocal communication between endothelial cells and pericytes during angiogenesis process (Gaengel et al., 2009). In the present study, we compared the expression of three angiogenic factors, VEGF, Ang-1 and Ang-2, after XSEC and EE treatment. Data in the present study demonstrated that XSEC and EE in combination robustly increased the VEGF protein expression which was coincident with the increased angiogenesis in the peri-infarct areas. And above all, combination treatment with XSEC and EE evoked a significant interactive effect on VEGF protein expression, meaning that the effects of the combination were synergistic. It should be noted that we did observe an increased expression of Ang-1 but a decreased expression of Ang-2 in EE, XSEC and XSEC+EE group, which was not significantly different compared to vehicle group. VEGF is the most prominent members of angiogenic growth factors, plays a critical role for proliferation, migration, and tube formation of endothelial cells (Zhao et al., 2017; Li Z. et al., 2018). Previous studies have shown that Ang-1 and Ang-2 depend on VEGF to modulate angiogenesis. Ang-1 as a pericyte-derived paracrine signal is essential for vessel maturation and stabilization (Valable et al., 2005; Yang et al., 2013). And Ang-2, an endogenous antagonist of Ang-1, induces vessel destabilization and participates in the initial phases of vessel sprouting during angiogenesis (Ma et al., 2013). Given that in the present study the expression of Ang-1 and Ang-2 were only evaluated on postoperative days 32, it cannot be excluded that the Ang-1 and Ang-2 levels may have been transiently altered but returned to normal following ischemic stroke. Thus, the dynamic changes of the post-stroke angiogenesis and the expression of angiogenic growth factors in the ischemic rats after XSEC and EE long-term treatment need to be further studied.

Middle cerebral artery occlusion typically results in extensive neuronal death in the cerebral striatum and cortex. Previous studies showed that cerebral ischemia can stimulate cell proliferation in the SVZ and redirect newly born cells migration to the ischemic striatum but not in the cerebral cortex of the adult brain (Arvidsson et al., 2002; Parent et al., 2002; Collin et al., 2005). Further studies have demonstrated that stroke induces cortical neurogenesis not only in animal models (Ortega et al., 2013), but also in adult human brain (Jin et al., 2006; Macas et al., 2006). In agreement with the above studies, our data indicated that increased Ki67-positive cells were located near the infarct border of striatum and cortex in MCAO rats. XSEC and EE, separately and in combination elevated the number of newly generated cells in the striatal and cortical ischemic boundary, however, only rats that received XSEC+EE treatment exhibited a significant augment of Ki67-positive cells in the peri-infarct cortical regions compared to control group rats. Neurogenesis involves the proliferation of neural progenitor cells as well as maintenance of the capacity of neuronal differentiation to repair damaged brain tissue (Bellenchi et al., 2013; Dai et al., 2013). Our data also suggested that the newly generated cells differentiated preferentially into neurons but less readily into GFAP-positive astrocytes in peri-infarct striatal and cortical regions after XSEC/EE treatment. In particular, combination treatment with XSEC and EE evoked a significant interactive effect in the astrocytic differentiation of newborn cells in the ischemic cortex after stroke. However, in contrast to the effect of XSEC and EE on down-regulation of Ki67-positive astrocytes in the damaged cortex, no such effect was seen in the ipsilateral striatum. In conclusion, the current study confirmed that combinatorial treatment provided greater benefits on the proliferation and differentiation of the neural progenitor cells than monotherapy with XSEC or EE. Several studies indicate that the microenvironments differ substantially between ischemic cortex and striatum, which may create their own cues to regulate of the proliferation and differentiation of the neural progenitor cells (Song et al., 2002; Zhang et al., 2005). Thus, our findings necessitate further investigation of the specific micro environmental factors that affect proliferation and differentiation of neural progenitor cells in the ischemic cortex and striatum, which may bring some new clues for studying the mechanism of XSEC/EE induced-neurogenesis following ischemic stroke.

Post-stroke angiogenesis has been proposed to be essential for neurogenesis, particularly under conditions of brain injury (Li Y. et al., 2018). Accumulating evidence demonstrates that axonal growth cones and endothelial tip cells are guided along the correct path via the coordinated action of attractive and repulsive axonal guidance cues from the external environment (Suchting et al., 2006), suggesting the potential involvement of axonal guidance signaling in the regulation of angiogenesis and neurogenesis. Data in the present study indicate a robust effect of combined XSEC and EE on up-regulating the protein expression levels of Netrin-1 and Robo-1 in the peri-ischemic brain tissues after stroke. Moreover, the combination of XSEC and EE evoked significant interactive effects in the Netrin-1and Robo-1 protein expression. It is noteworthy that Netrin-1 is the first of many neural guidance factors to induce axonal projection and function as a potent vascular mitogen (Finci et al., 2015). Netrin-1 stimulates endothelial cell proliferation and migration *in vitro* (Park et al., 2004), and induces angiogenesis and improves post-stroke neurovascular structure in adult mouse brains which may contribute to long-term functional outcome after ischemic injury (Fan et al., 2008; Sun et al., 2011; Lu et al., 2012). Similarly, the Slit-2/Robo-1 signaling pathway is initially characterized as a repulsive guidance cue for neuronal axons and neuronal migration during development (Borrell et al., 2012). Subsequently, evidence revealed that Slit-2 overexpression promoted critical endothelial processes required for angiogenesis such as endothelial cell proliferation, migration and tube formation through selective upregulation of Robo-1 (Wang et al., 2003; Li et al., 2015). These studies emphasized the vital roles of Netrin-1/DCC and Slit-2/Robo-1 in the regulation of angiogenesis and neurogenesis. In the present study, combination treatment of stroke showed a significant upregulation of Netrin-1 and Robo-1 levels expression as well as an increase of angiogenesis and neurogenesis in the ischemic brain after stroke, suggesting that axonal guidance signaling might be one of the underlying mechanisms involved in the beneficial effects of the combined actions of XSEC and EE on endogenous repair, e.g., angiogenesis and neurogenesis following cerebral ischemic injury.

There are several limitations of our study that should be mentioned. First, we only imaged rats at 31 days post-stroke, very little is known about the temporospatial neurovascular changes that occur during different stages of ischemic stroke. Future investigations designed to perform longitudinal MRI study of XSEC/EE-induced neurovascular restoration are very necessary. Second, in the present study, MRI and histological study were performed in rats without undergoing neurobehavioral experiments. Other MRI study have shown that rats underwent short periods of training in the MWM induced structural modifications and rapid changes in diffusion MRI indices (Hofstetter and Assaf, 2017). Similarly, MRI revealed mice trained on a spatial variant of the maze showed specific growth in the hippocampus, whereas mice trained on the cued version were found to have growth in the striatum. Especially, the structure-specific growth found furthermore correlated with GAP-43 staining (Lerch et al., 2011). In addition to the MRI study cited above, one study showed that MWM training on spatial memory acquisition enhanced the vesicular acetylcholine transporter expression in cholinergic neurons of rats (Engelmann et al., 2006). Although, we performed MRI and histological study in rats without neurobehavioral tests to avoid potential interaction between Morris water maze (MWM) testing and enriched environment. However, this introduces a second limitation in that we do not know the correlation between neurobehavioral function and MRI data. Further studies are required to demonstrated that neurovascular alterations are significantly correlated with functional recovery, which may be helpful in determining treatment outcomes during the recovery stage of ischemic stroke.

## Data Availability

All datasets generated for this study are included in the manuscript and/or the supplementary files.

## Ethics Statement

All experimental procedures were performed with protocols that were designed in accordance with the National Institutes of Health Guide for the Care and Use of Laboratory Animals, and approved by the Capital Medical University Animal Ethics Committee (Permit Number: AEEI-2018-052).

## Author Contributions

YZ carried out the research, analyzed the data, and wrote the manuscript. M-ZL performed the qRT-PCR experiments. X-FF prepared the samples. LY was responsible for rearing of animals. NZ carried out the behavioral tests. Y-YZ contributed to magnetic resonance imaging experiments. Q-XZ provided professional suggestions. HZ designed and supervised the study, and critically evaluated the final version of the manuscript.

## Conflict of Interest Statement

The authors declare that the research was conducted in the absence of any commercial or financial relationships that could be construed as a potential conflict of interest.

## Footnotes

1 https://www.slicer.org/
